# Agreement between Clinicians in Determining the Root Canal System in Radiographic Images

**Published:** 2007-01-20

**Authors:** Maryam Javidi, Mehdi Vatanpour, Shiva Shirazian

**Affiliations:** 1 *Department of Endodontics, Dental School, Mashad University of Medical Sciences, Mashad, Iran*; 2 *Department of Oral Medicine, Dental School, Mashad University of Medical Sciences, Mashad, Iran*

**Keywords:** Clearing Technique, Radiography, Root Canal Anatomy

## Abstract

**INTRODUCTION:** The aim of this study was to compare a clearing technique with conventional radiography in studying certain features of the root canal system in single root premolars. A secondary aim was to assess inter examiner agreement for these features using radiographs.

**MATERIALS AND METHODS:** Fifty-eight recently extracted single rooted premolars were included in this study. Two standard periapical radiographs were taken from buccolingual and 20° direction. The specimens were then decoronated, demineralized in 10% hydrochloric acid for 24 hours and then were cleared using methylsalicylate. The cleared teeth were examined using a magnifier (x10) and data relating to number of roots, canals, apical foramina and their positions were collected. The radiographs were examined by two independent trained endodontists using an X-ray viewer and the magnifying lens for same studied features using the clearing technique.

**RESULTS:** The kappa values for the agreement between the clearing technique and two examiners for the number of canals in standard radiographs were κ = 0.07, κ = 0.26 and in angulated radiographs were κ = 0.84, κ = 0.39 and κ = 0.31; for number of apical foramen were κ = 0.66, κ = 0.50, and κ = 0.19 and for detection the number of roots were %84 and %92 for examiner 1 and %92 and %88 for examiner 2.

**CONCLUSION:** In general, the kappa values were low to moderate for all comparisons. It is concluded that agreement between either radiographic examiners and clearing technique were poor to moderate indicating the limited value of radiographs alone at the time of studying certain aspects of the root canal system.

## INTRODUCTION

Successful root canal treatment depends on adequate clearing, shaping and filling of the root canal system. In order to achieve this, having detailed knowledge about root canal morphology of teeth is necessary for the operator (1).

Conventional radiography has traditionally been used in various stages of root canal treatment. Despite of demonstration of the main features, this radiography can not show the complexities of root canal anatomy. Previous studies have suggested that radiographic images are not reliable for detection of multiple canals (2) and lateral canals (3) and could not distinguish centrally placed apical foramina from those eccentrically located (4).

In general, discrepancies were found between *in vitro* and *in vivo* studies of root canal anatomy (5-6). Additionally, radiographs were also reported to be open to a wide range of interpretations in assessing the success of endodontic treatments (7-8).

The technique of clearing teeth has considerable value in studying the anatomy of the root canal system, because unlike radiographic images, it gives three dimensional view of the pulp cavity and allows a thorough examination of the pulp chambers and root canals (6, 9-12).

The clearing technique was also used in a study of apical leakage (13). However, the clearing technique remains useful only as a research tool with little or no clinical use. The aim of this investigation was to compare clearing technique with conventional radiography in studying certain features of root canal system. A secondary aim was to assess inter examiner agreement for these feature using radiographs.

**Figure 1:**
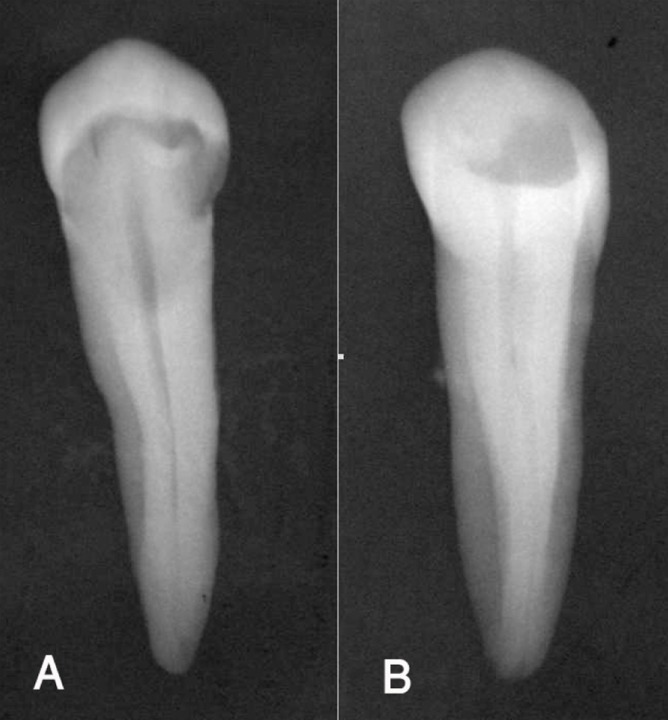
A: Straight, B: 20 degree angulated radiograph

## MATERIALS AND METHODS

Fifty-eight extracted single root premolars with intact root canal system were included in this investigation.

The extracted teeth were fixed in 10% normal saline placed in to small containers and labeled for identification. Each tooth was mounted on film using beading wax and oriented in a standard position by placing lingual surface on film in exact straight position. The first radiograph for each tooth was taken in a straight buccolingual direction (Figure 1A).

Then a second radiograph was taken with 20° mesially angulated direction like those taken in clinic (Figure 1B). The films used were Kodak Ekta speed (Eastman, Kodak, NY, USA) and were exposed for 0.25˝ using X-ray machine (Siemens, Germany), set at 65 kvp and 7.5 mA.

All teeth were exposed using a 16 cm cone, the end of which was standardized at 50mm from tooth. All exposed films were developed and fixed in an automatic processor (Velopex, England).

**Figure 2:**
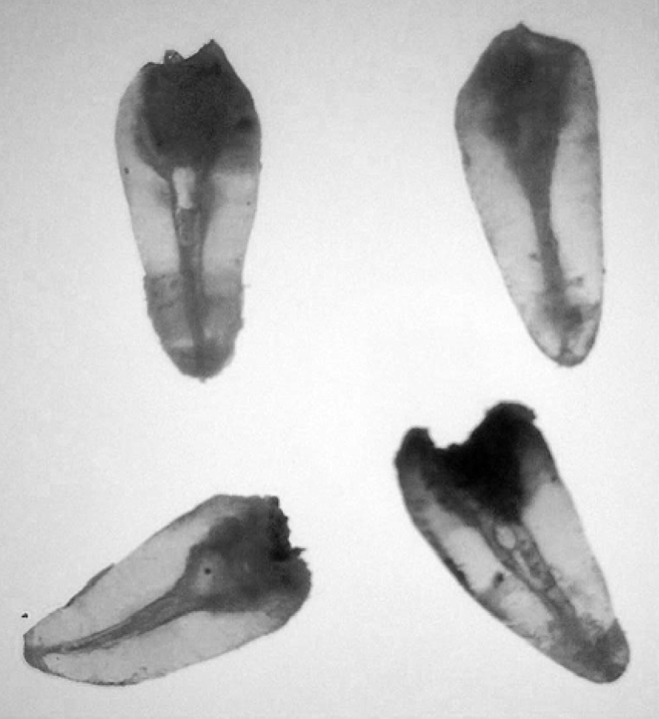
Cleared teeth

The teeth were decoronated and were placed in 5% sodium hypochlorite solution for 24h, and then were washed with running water and allowed to be dried for 24h. The teeth were then decalcified in 10% hydrochloric acid for 24h at room temperature and complete decalcification was verified by taking radiographs of teeth.

The teeth were then washed in running water for 24h. Dehydration was performed by placing the samples for 5h in each 70-100 percent ethyl alcohol. After dehydration the teeth were placed in 50% methylsalycilate for 5h and then in 100% concentration which rendered them transparent. Indian ink was used as contrast medium of the canals.

The cleared teeth (Figure 2) were examined by two independent trained endodontists using x-ray viewer and magnifier (x10). Data relating to number of roots, number of canals, position and number of apical foramina were collected using a standard format.

They were asked to collect data and complete questionnaire forms using similar format as the one used for cleared teeth.

All collected data were entered in a spread sheet of SPSS program. Kappa values for agreement of the presence or absence of root canal features between the outcomes of clearing technique examination compared to the radiographic examinations and between the two radiographic examiners were computed.

**Table 1:** Agreements between the clearing technique and two examiners in standard (S) and angulated (A) radiographs for number of roots (R), canals (C), and apical foramens (F) in percent

	**SR**	**AR**	**SC**	**AC**	**SF**	**AF**
E^a^1 - E2	52	63	45	84	66	73
E1 – G ^b^	84	92	07	39	50	19
E2 - G	92	88	26	31	19	02

***a:*** *Examiner* ***b:*** *Golden Standard or Clearing Technique*

**Table 2:** Agreement between the standard (S) and angulated (A) images for each examiner

	**SR/AR**	**SC/AC**	**SF/AF**
**Examiner 1 **	%71	%34	%66
**Examiner 2 **	%78	%63	%73

*R: number of roots, C: number of canals, F: number of apical foramens*

## RESULTS

Although all teeth included in this study were single root (as seen in clearing technique), either examiner 1 or 2 in straight and angulated radiographs were not in complete agreement with each other (Table 1). The kappa levels indicated a poor level of agreement between two examiners in straight and angulated radiographs (κ =0.25 and κ =0.63). Also, kappa value for each examiners between straight and angulated radiographs were moderate (κ =0.71 and κ =0.78) (Table 1).

Table 1 displays the results of agreement between two examiners (κ =0.45 and κ =0.84); and between the clearing technique and each of examiner 1 and 2 for number of canals found in radiographs (κ_1 _=0.7 & 0.39 and κ_2 _= 0.26 & 0.31). These results show that all of agreements were poor and only in detection of number of canals between two examiners a good agreement was observed (κ =0.84) (Table 1). Examiner 1 has poor agreement in finding the number of canals in standard and angulated radiographs (κ =0.34) and the kappa value for examiner 2 in standard and angulated radiograph was 0.63 (Table 1).

As seen in Table 2, the agreement between two examiners for explaining the number of apical foramen was moderate (κ=0.66 and κ=0.73) also the agreement between these examiners and the clearing technique either in standard radiographs (κ_1 _=0.5 and κ_2 _=0.19) or in angulated radiographs (κ_1 _=0.19 and κ_2 _= 0.02) were poor. Table 1 display that for two (both) examiners the agreement between standard and angulated radiographs were moderate (κ_1 _= 0.66 and κ_2 _=0.77).

## DISCUSSION

Conventional radiography is commonly used in various stages of endodontic treatment. A radiographic image is a two dimensional representation for a three-dimensional object and it is open to a wide range of interpretation (7).

The dental X-ray films used in this study (Kodak D-speed, Eastman Kodak, NY, USA) were chosen because of their routinely use in endodontic clinics while they have also higher resolution and provide more contrast (15).

The agreement between the results of clearing technique and either radiographic examiner was mostly low. In general, the kappa values were poor to moderate in all comparisons and this means that none of radiographic examiners were reliable for explaining the morphological characteristics determined using clearing technique.

Of all the studied features, the least agreement between two examiners and the clearing technique appeared to be in relation with the number of apical foramen in angulated radiographs for examiner 2(κ =0.31), and to the number of apical foramen in standard radiograph for examiner 1(κ =0.50).

It was also of interest to note the relatively poor agreement between two examiners for all variables compared.

The least agreement between two examiners was in detecting the number of canals in standard radiographs (κ =0.45) and the highest agreement was detected for the number of canals in angulated radiographs.

The results of this study showed that the inability of radiographic technique and poor interpretation of root canal morphology are resulting from deficiency of the technique albeit using different tooth.

Morphotypes and study designs have, in general, reported similar limitations of radiographs in studying certain features of the root canal system (2,3,7,8,15). It is important to note that the clearing technique is a research tool and has little or no clinical applicability. However, despite of the importance of radiographs in clinical endodontics, the limitations in defining certain aspect of root canal anatomy are noteworthy.

## CONCLUSION

It is concluded that in most cases, the agreement between two examiners and also each of them and the clearing technique were, poor to moderates. This indicates some limitation in the value of radiographs alone in describing aspects of root canal system. However, the clearing technique remains useful only as a research tool with little or no clinical applicability.
